# Effects of Silage Diet on Meat Quality through Shaping Gut Microbiota in Finishing Pigs

**DOI:** 10.1128/spectrum.02416-22

**Published:** 2022-12-12

**Authors:** Jiakuan Niu, Xiao Liu, Junying Xu, Fen Li, Jincan Wang, Xixi Zhang, Xu Yang, Lin Wang, Sen Ma, Defeng Li, Xiaoyan Zhu, Chengzhang Wang, Yinghua Shi, Yalei Cui

**Affiliations:** a College of Animal Science and Technology, Henan Agricultural University, Zhengzhou, Henan, China; b Henan Key Laboratory of Innovation and Utilization of Grassland Resources, Zhengzhou, Henan, China; c Henan Forage Engineering Technology Research Center, Zhengzhou, Henan, China; USDA—ARS

**Keywords:** finishing pigs, meat quality, mulberry silage, paper mulberry silage, SCFAs, gut microbiota

## Abstract

With increasing demand for high-quality pork, development of green and healthy feed for finishing pigs is urgently needed. In this study, the effects and mechanisms of mulberry and paper mulberry silages on growth performance, meat quality, and intestinal health of finishing pigs were explored. Intestinal microbiota were profiled, and microbially produced short-chain fatty acids (SCFAs) were measured. The average daily gain (ADG) and feed conversion rate (FCR) with mulberry and paper mulberry silages were not significantly different from those of the control. Meat quality as measured by pork marbling and fatty acids in the longissimus dorsi was better with mulberry silage. The highest concentration of SCFAs was also with mulberry silage. According to 16S rRNA sequencing, *Clostridium_sensu_stricto_1*, *Terrisporobacter*, and *Lachnospiraceae*, which are important in SCFA production, were biomarkers of mulberry silage. PICRUSt functional analysis of intestinal microbes indicated that galactose metabolism, starch and sucrose metabolism, and carbohydrate digestion and absorption decreased significantly in silage treatments but increased in the control. Correlations between intestinal microbes and SCFAs and fatty acids indicated *Clostridium_sensu_stricto_1*, *Terrisporobacter*, and *Lachnospiraceae* were closely associated with SCFA and fatty acid contents. The results indicated that mulberry silage could increase SCFA content through shaping intestinal microbes to affect the deposition of fatty acids, which laid a solid theoretical foundation for improving pork quality.

**IMPORTANCE** To avoid competition between people and animals for food, it is essential to develop nontraditional feeds. In this study, the effects of the silages of the unconventional feed resources mulberry and paper mulberry on meat quality of finishing pigs were examined. With mulberry silage in the diet, meat quality improved as indicated by meat color, marbling score, and beneficial fatty acids in the longissimus dorsi muscle. Pigs fed mulberry silage had the highest concentrations of short-chain fatty acids (SCFAs), and 16S rRNA sequencing identified *Clostridium_sensu_stricto_1*, *Terrisporobacter*, and *Lachnospiraceae* as biomarkers, which are important in SCFA production. Functions of intestinal microbes in the two silage groups primarily involved amino acid metabolism and SCFA production. Correlations between intestinal microbes and SCFAs and fatty acids indicated that *Clostridium_sensu_stricto-1*, *Terrisporobacter*, and *Lachnospiraceae* were closely associated with SCFA contents in the intestine and fatty acids in the longissimus dorsi.

## INTRODUCTION

Pork is one of the most important meat products for consumers. As the living standard of people improves, the demand for high-quality pork increases. Many factors affect pork quality, among which feed is the most important and the most easily controlled (1). Competition between people and animals for food is currently a serious problem. Therefore, there is demand to develop nontraditional feeds with potential benefits for animal productivity. Grasses, shrubs, trees, and other resources have been widely explored as nontraditional feeds to provide a high-protein and -fiber supplement to low-protein and -fiber feed. Mulberry and paper mulberry are unconventional feed resources. The crude protein content of mulberry leaves is 27.63 to 37.36 g/100 g dry weight (DW), the crude fiber content is 9.90 to 13.85 g/100 g DW (2), and the crude fat content is 0.64% to 1.51% (3). Mulberry is currently widely used in livestock and poultry production (4). Mulberry leaves also contain a variety of bioactive substances, such as alkaloids, polysaccharides, and flavonoids (5), which have antioxidant and antibacterial activities and improve immunity (6). Therefore, mulberry leaves are considered “green and healthy” feed additives, and when used properly, they can replace probiotics to improve animal immune function and prevent diseases (7). Mulberry leaves have good palatability and high digestibility—generally as high as 70% to 90%—with relatively high protein and essential amino acid contents, which is very similar to alfalfa hay (8). Because of such characteristics, mulberry leaves have recently become a focus of research as an unconventional feed for animals. Paper mulberry is another tree in the family Moraceae with leaves that are rich in nutrients and contain high crude protein, crude ash, crude fat, and phosphorus contents and a suitable content of crude fiber (9). Paper mulberry leaves contain 21.6% crude protein, 4.3% ether extract, and 1.9% calcium (10). The leaves are usually used as a type of high-quality feed for cattle, pigs, and sheep because of the high nutritional value and high contents of crude protein, lysine, and methionine. Because of the many beneficial effects, including antibacterial and antioxidative activities and increasing immunity, paper mulberry leaves are also often used as animal feed supplements (11). Paper mulberry leaves are usually made into silage to improve digestibility. Addition of paper mulberry leaves extract to food for weaned piglets at an appropriate dose can improve growth performance, increase antioxidant capacity and immune function, and reduce diarrhea (12).

According to their own characteristics, mulberry and paper mulberry can be made into silage feed for animals, which has many advantages. Silage reduces nutrient loss during harvest and storage to the maximum extent and improves the efficiency of feed treatment (13). Silage feed has high moisture content and good palatability, which can eliminate antinutritional factors (14). However, 50% of protein is converted into nonprotein nitrogen in silage feed, which cannot be used by monogastric animals. Recent studies have shown that intestinal microbes can regulate host-related biological processes, including nutrient production and energy metabolism in diet (15). Moreover, a study has shown that the external environment and feed are the main factors affecting gut microbes (16). Therefore, adjustment of intestinal microbes through feed to improve animal growth performance is an effective strategy. Many studies are currently investigating silage application in monogastric animals. For example, feeding fattening pigs fermented apple pomace can affect meat quality and fatty acid composition (17), and feeding broilers a fermented cottonseed meal diet has positive effects on growth performance and intestinal health (18). However, the application of mulberry and paper mulberry silages (MS and PMS, respectively) in pig production needs further study.

In this study, to investigate the effects of MS and PMS on finishing pigs, growth performance, meat quality, intestinal microbes, and short-chain fatty acids (SCFAs) in the intestine were analyzed. In addition, mechanisms were explored by examining interactions between intestinal microbes, SCFAs, and muscle fatty acids. The goal was to clarify the influence and possible mechanisms of MS and PMS feeds on growth performance, meat quality, and intestinal health of finishing pigs, as well as provide a solid theoretical foundation to improve pork quality and strategies to identify new feed resources.

## RESULTS

### Effects of mulberry and paper mulberry silages on growth performance of finishing pigs.

To investigate the effects of mulberry and paper mulberry silages (MS and PMS, respectively) on growth performance of finishing pigs, the average daily gain (ADG) and feed conversion rate (FCR) of finishing pigs in different treatment groups were calculated. Each treatment had 4 replicates, and each replicate had 20 finishing pigs. The ADG and FCR of mulberry and paper mulberry silage groups were not significantly different from those of the control (*P* > 0.05) (Table 1).

**TABLE 1:** Effects of different diets on growth performance of finishing pigs

Group^*a*^	Result for^*b*^:	
	ADG (kg/head/day)	FCR
CON	0.87 ± 0.06	3.12 ± 0.21
MS	0.89 ± 0.02	3.05 ± 0.07
PMS	0.82 ± 0.08	3.31 ± 0.29

a CON, control group; MS, mulberry silage group; PMS, paper mulberry silage group.

b ADG, average daily gain; FCR, feed conversion rate. The data were evaluated by one-way ANOVA, and the difference between the average values was evaluated by Duncan’s test. *P* < 0.05 is considered to be statistically significant. The values are the mean ± standard deviation. Each treatment has 4 replicates, and each replicate has 20 finishing pigs (*n* = 80).

### Effects of mulberry and paper mulberry silages on meat quality.

**FIG 1:**
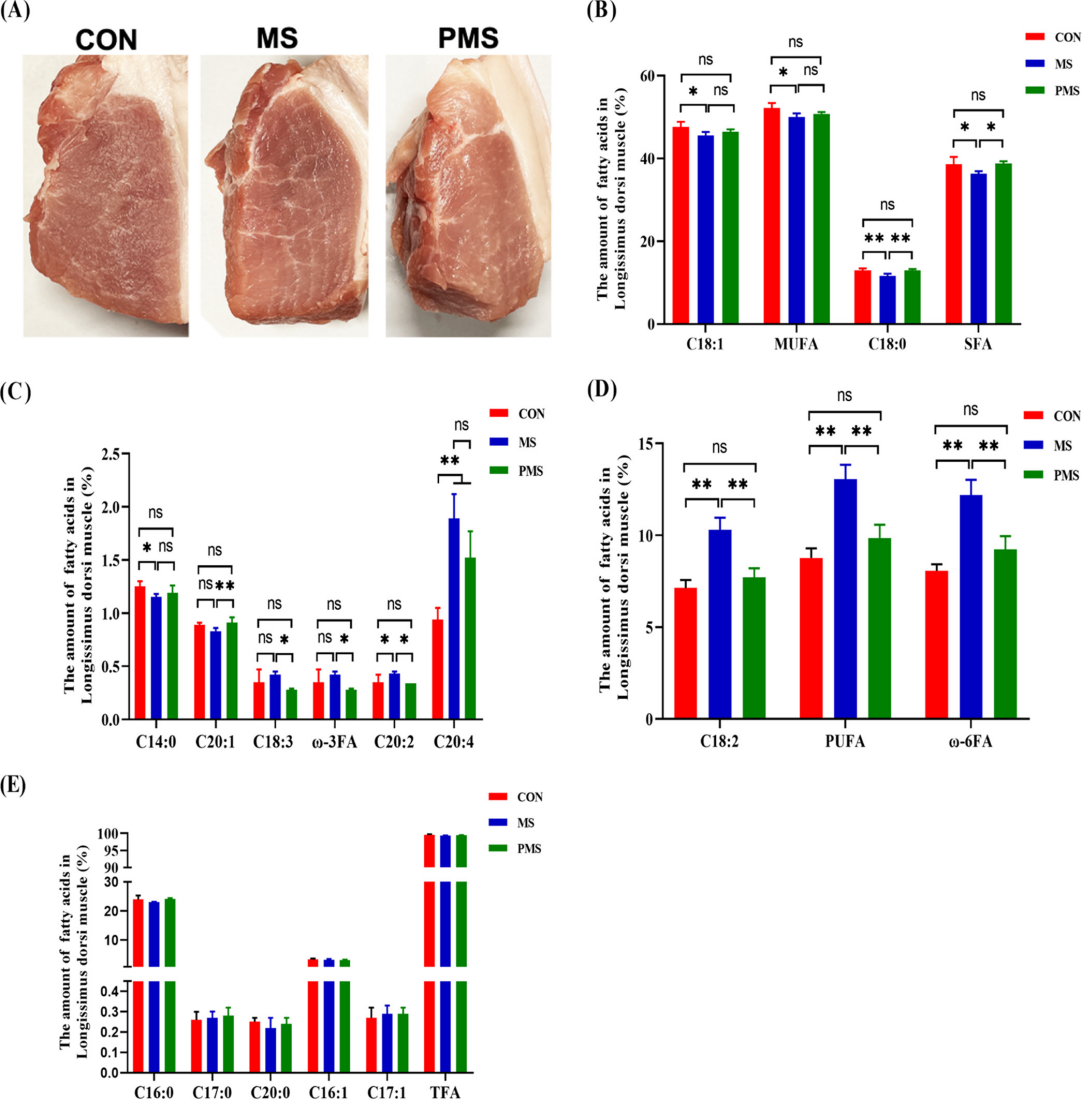
Effects of mulberry and paper mulberry silages on meat quality and fatty acids in the longissimus dorsi muscle of finishing pigs. (A) Marbling score of pork; (B to E) content of fatty acids in longissimus dorsi muscle. CON, control; MS, mulberry silage; PMS, paper mulberry silage. One sample was randomly selected from each replicate in the same treatment (i.e., 4 samples per treatment [*n* = 4]). The data were evaluated by one-way ANOVA, and the difference between the average values was evaluated by Duncan’s test (*, *P* < 0.05; **, *P* < 0.01; ns, nonsignificant difference). The error bars show the standard deviation (SD).

To determine the effects of mulberry and paper mulberry silages on the physical characteristics of meat, meat color, water holding capacity, marbling score, and drip loss were observed in 4 samples per treatment. One sample was randomly selected from each replicate in the same treatment. Meat color was assessed by redness (a*), yellowness (b*), and lightness (L*). The L* value is the brightness coefficient, and a* and b* values are the color coefficients for red-green and yellow-blue, respectively. The L* value of the paper mulberry silage group was significantly higher than that of the control, whereas there was no significant difference between silage groups (Table 2). The a* value in the mulberry silage group tended to increase over that of the control group. The water holding capacity of the paper mulberry silage group was significantly higher than those of the control and mulberry silage groups (*P* < 0.01) (Table 2). There was no significant difference in marbling scores between silage groups, but both had significantly higher scores than that in the control (*P* < 0.05) (Table 2). Meat marbling images with the three treatments are shown in Fig. 1A.

**TABLE 2:** Effects of the different diets on meat quality of finishing pigs

Characteristic	Result for^*a*^:		
	CON	MS	PMS
Lightness (L*)	49.72 ± 0.52 b	50.28 ± 1.14 ab	51.31 ± 0.86 a
Redness (a*)	9.55 ± 1.37	10.15 ± 0.41	9.30 ± 0.80
Yellowness (b*)	2.73 ± 2.47	1.82 ± 0.20	1.89 ± 0.77
Drip loss (%)	4.20 ± 0.36	3.33 ± 0.92	3.67 ± 1.46
Water holding capacity (%)	2.80 ± 0.10 B	3.10 ± 0.35 B	4.33 ± 0.40 A
Marbling score	1.83 ± 0.29 b	2.33 ± 0.29 a	2.50 ± 0.00 a

a CON, control group; MS, mulberry silage group; PMS, paper mulberry silage group. The data were evaluated by one-way ANOVA, and the difference between the average values was evaluated by Duncan’s test. Values are the mean ± standard deviation. Different small letters indicate significant difference (*P *< 0.05), and the presence of the same small letters or no letters indicates no significant difference (*P* > 0.05). Different capital letters indicate extremely significant difference (*P* < 0.01), and the presence of the same capital letters or no letters indicates no significant difference (*P* > 0.05). One sample was randomly selected from each replicate in the same treatment (i.e., 4 samples per treatment [*n* = 4]).

Among fatty acids of the longissimus dorsi muscle, oleic acid (C_18:1_), monounsaturated fatty acid (MUFA), and myristic acid (C_14:0_) decreased significantly in the MS group compared with the control (*P* < 0.05) (Fig. 1B and C). In addition, stearic acid (C_18:0_) and saturated fatty acid (SFA) decreased significantly in the MS group compared with the control and PMS groups (*P* < 0.01) (Fig. 1B). Compared with the PMS group, arachidonic acid (C_20:1_) decreased significantly in the MS group (*P* < 0.01) (Fig. 1C), whereas α-linolenic acid (C_18:3_) and ω-3FA increased significantly in the MS group (*P* < 0.05) (Fig. 1C). Arachidonic acid (C_20:4_) increased significantly in the MS and PMS groups (*P* < 0.01) (Fig. 1C). Arachidonic acid (C_20:2_), linoleic acid (C_18:2_), polyunsaturated fatty acid (PUFA), and ω-6FA increased significantly in the MS group compared with the control and PMS groups (Fig. 1C and D). There were no significant differences in amounts of other fatty acids among the three groups (Fig. 1E). The results suggested that the mulberry silage treatment was the most beneficial for the production of fatty acids in the longissimus dorsi.

### Concentrations of short-chain fatty acids in the colon of finishing pigs.

**FIG 2:**
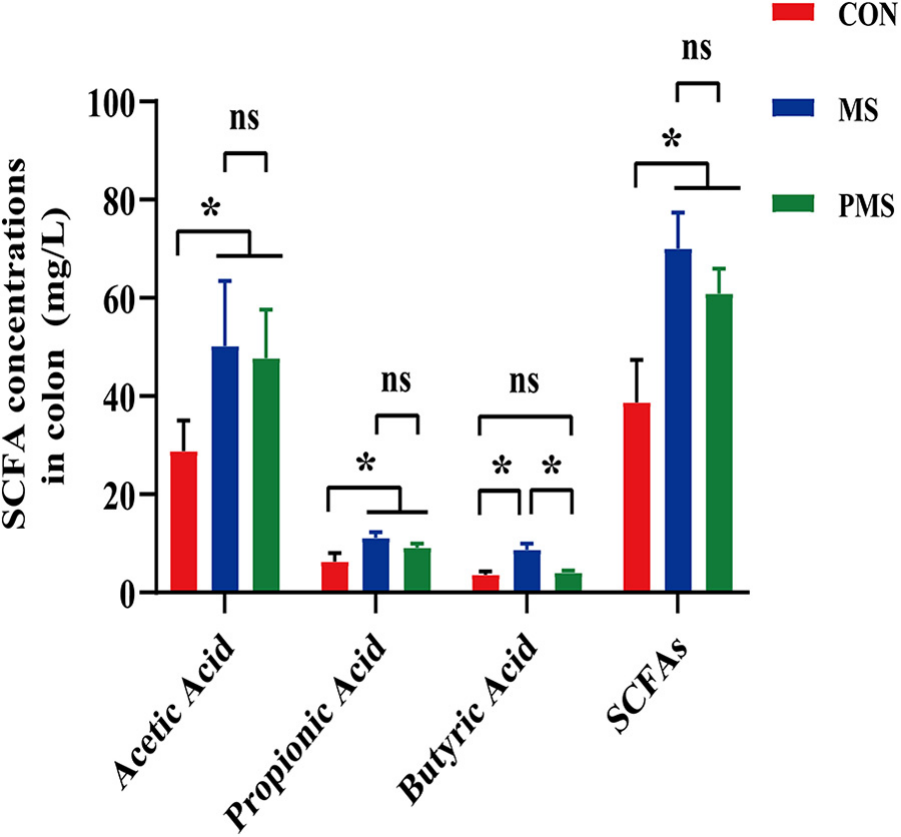
Concentration of short-chain fatty acids in the colon of finishing pigs. CON, control; MS, mulberry silage; PMS, paper mulberry silage. One sample was randomly selected from each replicate in the same treatment (i.e., 4 samples per treatment [*n* = 4]). The data were evaluated by one-way ANOVA, and the difference between the average values was evaluated by Duncan’s test (*, *P* < 0.05; **, *P* < 0.01; ns, nonsignificant difference). The error bars show the SD.

One sample was randomly selected from each replicate in the same treatment (i.e., four samples per treatment), and concentrations of SCFAs in the colon of finishing pigs were determined. Concentrations of acetic acid (AA), propionic acid (PA), and total SCFAs in the two silage groups were significantly higher than those in the control (*P* < 0.05) (Fig. 2). Butyric acid (BA) content was significantly higher in the mulberry silage group than in the paper mulberry silage and control groups (*P* < 0.05) (Fig. 2).

### Diversity index analysis of cecum and colon microbes.

Four cecum samples and four colon samples per treatment (one sample was randomly selected from each replicate in the same treatment, and cecum and colon samples were from the same pig) were sequenced by Illumina MiSeq, and 1,489,912 optimized sequences were obtained, with a total of 646,921,498 bases. The average sequence length of the samples was ~434 bp. Totals of 1,133 operational taxonomic units (OTUs) in the cecum and 1,165 OTUs in the colon were obtained. Rarefaction curves of deep sequencing of the 16S rRNA gene in the V3-V4 region of cecum samples are presented in Fig. S1A in the supplemental material. In the cecum, there were 1,133 OTUs in the three treatment groups. There were 786 OTUs in the three treatment groups, 43 OTUs in the cecum control (CCe) and mulberry silage (MSCe) groups, 66 OTUs in the CCe and cecum paper mulberry silage (PMSCe) groups, and 98 OTUs in the MSCe and PMSCe groups (Fig. S1B). Principal-coordinate analysis (PCoA) indicated the cecum microbial compositions of the MSCe and PMSCe treatment groups were similar (Fig. S1C). Richness and diversity of cecum microbes in different treatment groups were also analyzed. There were no significant differences in the Shannon and Chao indices among cecum microbes in different treatments, but diversity and richness of microbes in the two silage groups were higher than those in the control (Fig. S1D and E). Rarefaction curves of deep sequencing of the 16S rRNA gene in the V3-V4 region of cecum samples are presented in Fig. S2A. In the colon, there were 1,165 OTUs in the three treatment groups. There were 831 OTUs in three treatment groups, 41 OTUs in the colon control (CCo) and mulberry silage (MSCo) groups, 67 OTUs in the CCo and colon paper mulberry silage (PMSCo) groups, and 90 OTUs in the MSCo and PMSCo groups (Fig. S2B). A PCoA indicated that microbes in the MSCo and PMSCo treatment groups were similar and were separate from those in the CCo treatment group (Fig. S2C). There were no significant differences in the Shannon index results among treatments. However, the Chao index of the PMSCo group was significantly higher than that of the control, indicating that species richness in the PMSCo group was significantly higher than that of the control (*P* < 0.05) (Fig. S2D and E).

### Composition and difference in intestinal microbes at the phylum level.

**FIG 3:**
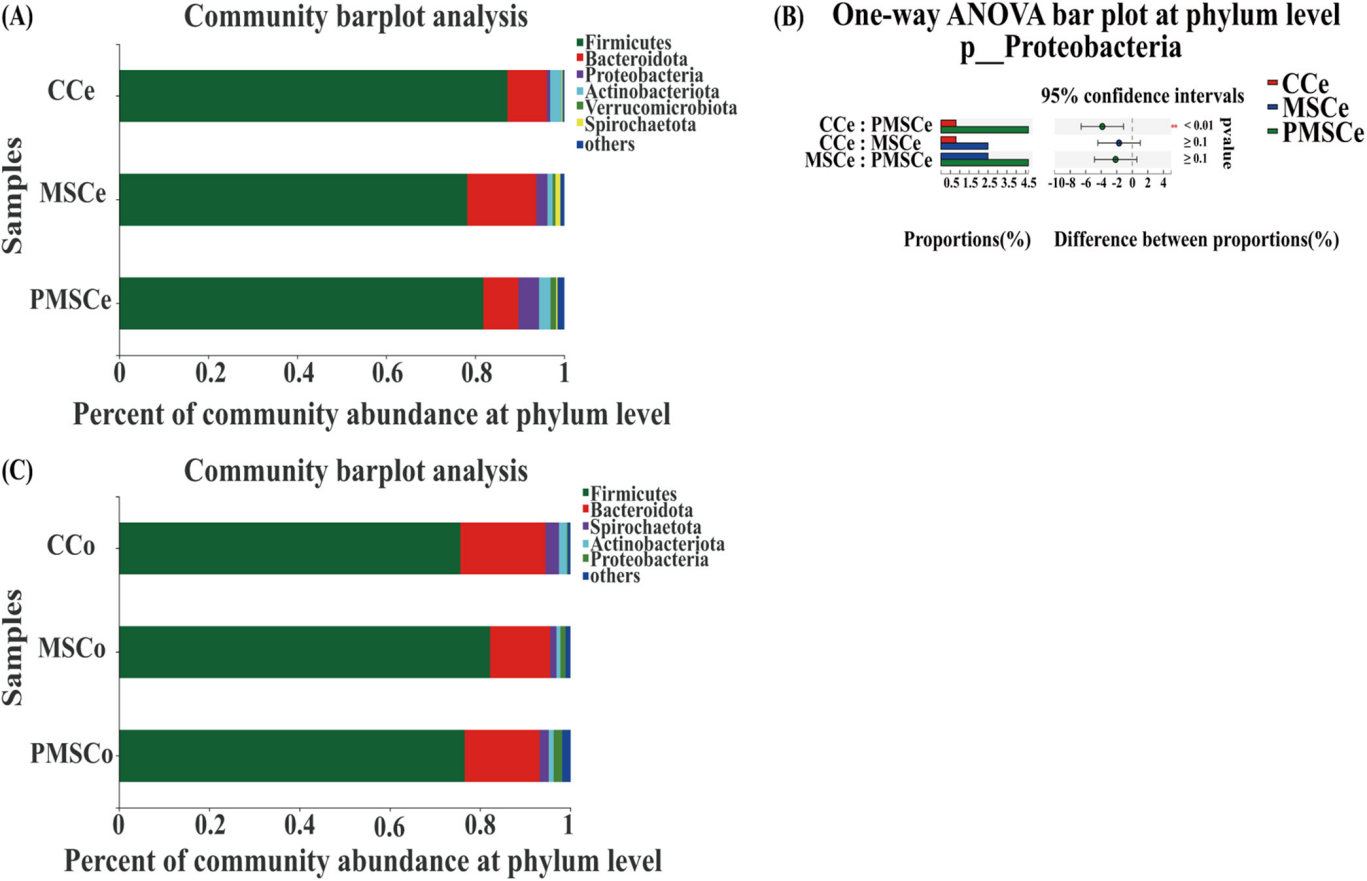
Composition of and differences in intestinal microbes at the phylum level. (A) Microbial composition of the cecum at the phylum level; (B) differential expression of microbes in different groups; (C) microbial composition of the colon at the phylum level. The data are means ± SD. *, 0.01 < *P* < 0.05; **, *P* < 0.01. CCe, cecum of the control; MSCe, cecum of the mulberry silage group; PMSCe, cecum of the paper mulberry silage group; CCo, colon of the control; MSCo, colon of the mulberry silage group; PMSCo, colon of the paper mulberry silage group. One sample was randomly selected from each replicate in same treatment (i.e., 4 samples in cecum and 4 samples in colon per treatment [*n* = 4]).

Cecum microbes were mainly composed of six phyla, including *Firmicutes*, *Bacteroidetes*, *Actinobacteria*, *Proteobacteria*, *Verrucomicrobia*, and *Spirochaetae* (Fig. 3A). *Firmicutes* and *Bacteroidota* were the two most important phyla, accounting for more than 88% in the three groups. The five most abundant phyla of bacteria were *Firmicutes* (87.27%), *Bacteroidota* (8.80%), *Actinobacteriota* (2.49%), *Proteobacteria* (0.80%), and *Spirochaetota* (0.27%) in the CCe group, *Firmicutes* (78.23%), *Bacteroidota* (15.53%), *Proteobacteria* (2.50%), *Actinobacteriota* (1.16%), and *Spirochaetota* (1.11%) in the MSCe group, and *Firmicutes* (81.86%), *Bacteroidota* (7.85%), *Proteobacteria* (4.65%), *Actinobacteriota* (2.57%), and *Verrucomicrobiota* (1.26%) in the PMSCe group (Fig. 3A). The abundance of *Proteobacteria* in both silage groups was higher than that in the control group, but only in the PMSCe group was the abundance significantly higher than that in the control group (*P* < 0.01) (Fig. 3B).

The composition of the colon microbe phyla was similar to that in the cecum (Fig. 3C), with *Firmicutes*, *Bacteroidetes*, *Spirochaetae*, *Actinobacteria*, and *Proteobacteria* the most abundant phyla. *Firmicutes* and *Bacteroidota* were the two most important phyla, accounting for more than 92% in the three groups. The five most abundant phyla of bacteria were *Firmicutes* (75.65%), *Bacteroidota* (18.84%), *Spirochaetota* (2.99%), *Actinobacteriota* (1.74%), and *Desulfobacterota* (0.31%) in the CCo group, *Firmicutes* (82.21%), *Bacteroidota* (13.35%), *Spirochaetota* (1.35%), *Proteobacteria* (1.14%), and *Actinobacteriota* (0.91%) in the MSCo group, and *Firmicutes* (76.58%), *Bacteroidota* (16.63%), *Spirochaetota* (2.02%), *Proteobacteria* (1.88%), and *Actinobacteriota* (1.08%) in the PMSCo group (Fig. 3C). However, there were no significant differences in phyla of colon microbes among the groups.

### Screening of cecum microbial biomarkers at the genus level.

**FIG 4:**
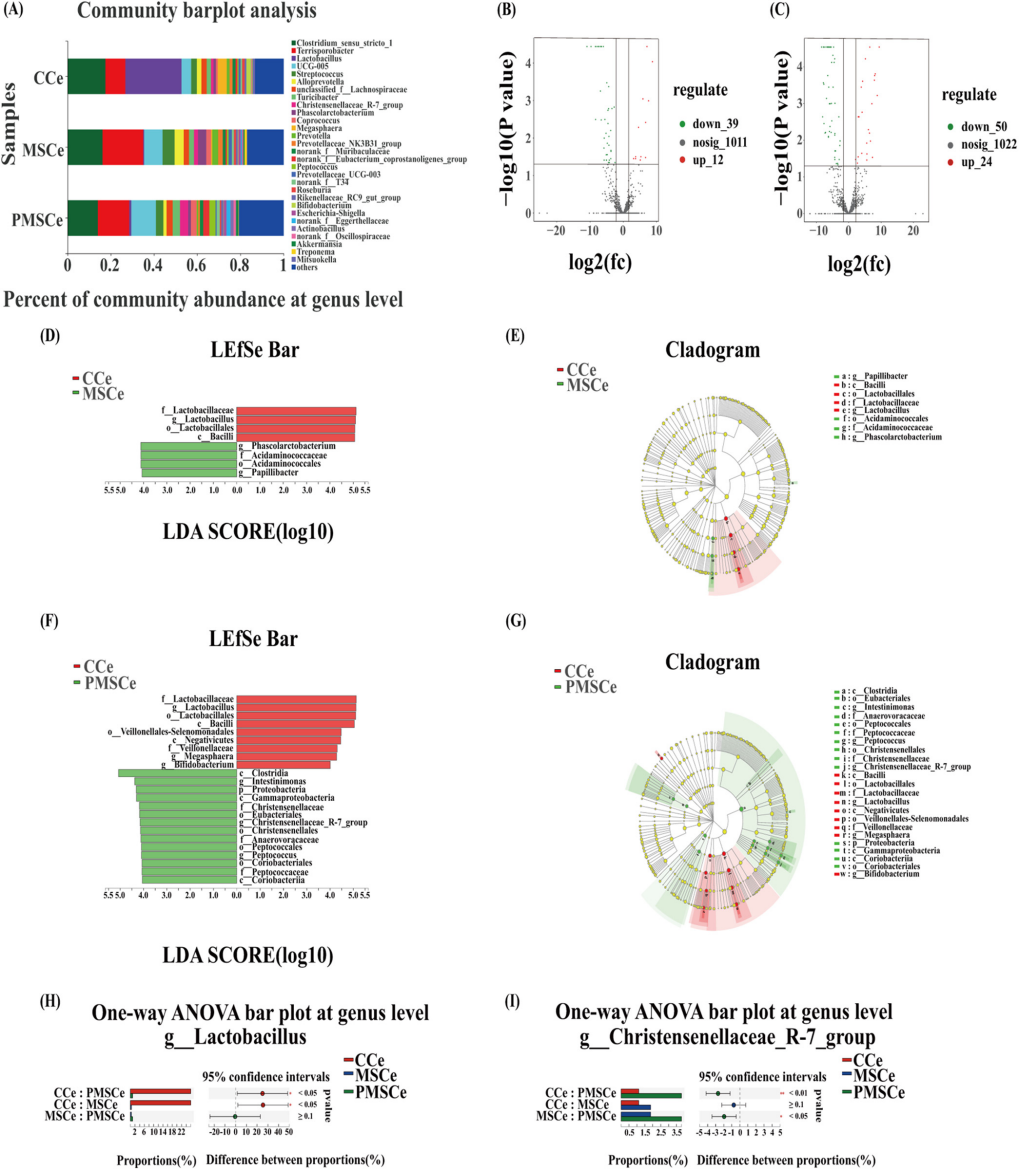
Screening of microbial biomarkers of the cecum at the genus level. (A) Differential analysis of cecum microbes at the genus level; (B and C) differential volcano map (*x-*axis coordinate, log_2_ fold change [FC]; *y-*axis coordinate, adjusted *P* value). Each point in the graph represents an operational taxonomic unit (OTU), and the two lines parallel to the *y* axis represent FC = 2 and FC = −2. The dotted line parallel to the *x* axis represents −log_10_ (0.05), and the points above the dotted line represent OTUs with significance at *P* < 0.05. For each OTU, when *P* is <0.05 and at an FC of ≥2, the OTU has an intergroup difference. (D and F) Linear discriminant analysis (LDA) effect size (LEfSe) analysis representing differentially abundant taxa in the cecum (CCe and MSCe and CCe and PMSCe); (E and G) LDA cladogram (CCe and MSCe and CCe and PMSCe); (H and I) differential expression of intestinal microbes: (H) *Lactobacillus* and (I) *Christensenellaceae_R-7_group*. Values are the mean ± SD. *, 0.01 < *P* < 0.05; **, *P* < 0.01. CCe, cecum of the control; MSCe, cecum of the mulberry silage group; PMSCe, cecum of the paper mulberry silage group. There were 4 samples in cecum per treatment (*n* = 4).

Cecum microbial communities were relatively complex, with many species. The three most abundant genera of microbes in the cecum were *Lactobacillus* (25.95%), *Clostridium_sensu_stricto_1* (17.56%), and *Terrisporobacter* (9.25%) in the CCe group, *Terrisporterobacter* (18.83%), *Clostridium_sensu_stricto*_1 (16.21%), and *UCG-005* (8.52%) in the MSCe group, and *Terrisporobacter* (14.57%), *UCG-00*5 (11.42%), and *Clostridium_sensu_stricto_1* (14.03%) in the PMSCe group (Fig. 4A). The volcano map showed that compared with CCe, 39 OTUs were significantly downregulated and 12 were significantly upregulated in the MSCe group (Fig. 4B) and 50 OTUs were significantly downregulated and 24 were significantly upregulated in the PMSCe group (Fig. 4C).

On the basis of results of the volcano map, a linear discriminant analysis (LDA) effect size (LEfSe) analysis was used to identify the biomarkers associated with the silage groups. Candidate biomarkers in the MSCe group included *Phascolarctobacterium*, *Acidaminococcaceae*, *Acidaminococcales*, and *Papillibacter* (Fig. 4D and E), and those in the PMSCe group included *Clostridia*, *Intestinimonas*, *Proteobacteria*, *Gammaproteobacteria*, *Christensenellaceae*, *Eubacteriales*, *Christensenellaceae_R-7_group*, *Christensenellales*, *Anaerovoracaceae*, *Peptococcales*, *Peptococcus*, *Coriobacteriales*, *Peptococcaceae*, and *Coriobacteriia* (Fig. 4F and G). Thus, those microbial species were closely associated with the two silage groups. Relative abundance of *Lactobacillus* in the two silage groups decreased significantly compared with that in the CCe group (*P* < 0.05) (Fig. 4H). The relative abundance of *Christensenellaceae_R-7_group* in the PMSCe group increased significantly compared with those in the CCe and MSCe groups (Fig. 4I), indicating that the members of *Christensenellaceae_R-7_group*, acting as differential core microbes, played important roles in PMSCe group.

### Screening of colon microbial biomarkers at the genus level.

**FIG 5:**
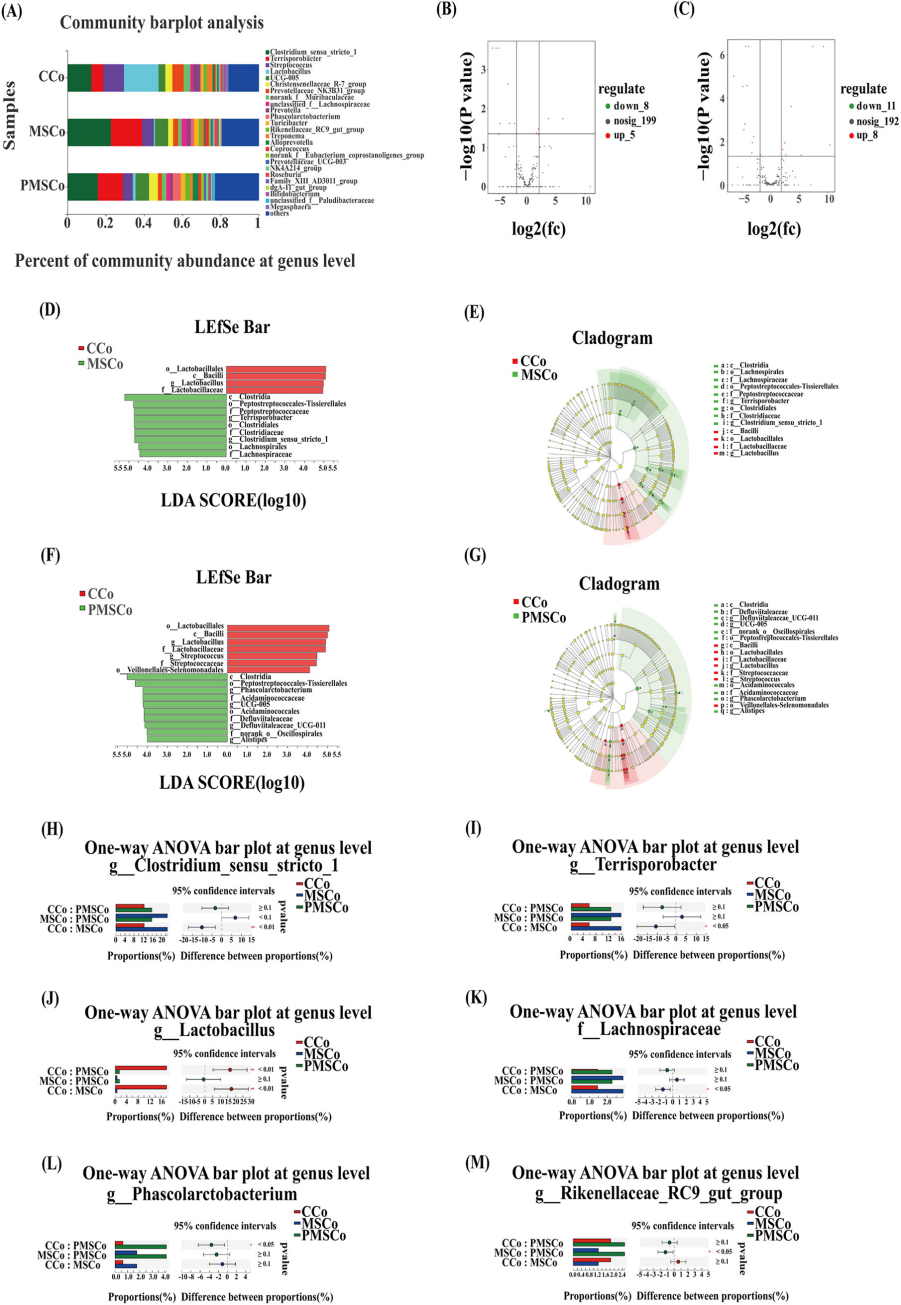
Screening of microbial biomarkers of the colon at the genus level. (A) Differential analysis of colon microbes at the genus level; (B and C) differential volcano map (*x-*axis coordinate, log_2_ fold change [FC]; *y-*axis coordinate, adjusted *P* value). Each point in the graph represents an operational taxonomic unit (OTU), and the two lines parallel to the *y* axis represent FC = 2 and FC = −2. The dotted line parallel to the *x* axis represents −log_10_ (0.05), and the points above the dotted line represent OTUs with significance at *P* < 0.05. For each OTU, when *P* is <0.05 and at an FC of ≥2, the OTU has an intergroup difference. (D and F) Linear discriminant analysis (LDA) effect size (LEfSe) analysis representing differentially abundant taxa in the colon (CCo and MSCo and CCo and PMSCo); (E and G) LDA cladogram (CCo and MSCo and CCo and PMSCo); (H to M) differential expression of intestinal microbes: (H) *Clostridium_sensu_stricto_1*, (I) *Terrisporobacter*, (J) *Lactobacillus*, (K) *Lachnospiraceae*, (L) *Phascolarctobacterium*, and (M) *Rikenellaceae_RC9_gut group*. Values are means ± SD. *, 0.01 < *P* < 0.05; **, *P* < 0.01. CCo, colon of the control; MSCo, colon of the mulberry silage group; PMSCo, colon of the paper mulberry silage group. There were 4 samples in colon per treatment (*n* = 4).

The three most abundant genera of microbes in the colon were *Lactobacillus* (17.96%), *Clostridium_sensu_stricto_1* (12.47%), and Streptococcus (10.86%) in the CCo group, *Clostridium_sensu_stricto_1* (22.69%), *Terrisporter* (16.35%), and *UCG-005* (6.84%) in the MSCo group, and *Clostridium_sensu_stricto_1* (15.85%), *Terrisporobacter* (13.14%), and *UCG-005* (7.00%) in the PMSCo group (Fig. 5A). The volcano map showed that compared with the CCo group, 8 OTUs were significantly downregulated and 5 were significantly upregulated in the MSCo group (Fig. 5B) and 11 OTUs were significantly downregulated and 8 were significantly upregulated in the PMSCo group (Fig. 5C).

On the basis of the significantly different species screened at the genus level, an LEfSe analysis was used to identify colon biomarkers associated with the two experimental treatments. There were significant differences in relative abundances at the genus level between the control and the two silage groups. Compared with the control, candidate biomarkers in the MSCo group included *Clostridia*, *Peptostreptococcales-Tissierellales*, *Peptostreptococcaceae*, *Terrisporobacter*, *Clostridiales*, *Clostridiaceae*, *Clostridium_sensu_stricto_1*, *Lachnospirales*, and *Lachnospiraceae* (Fig. 5D and E) and those in the PMSCo group included *Clostridia*, *Peptostreptococcales-Tissierellales*, *Phascolarctobacterium*, *Acidaminococcaceae*, *UCG-005*, *Acidaminococcales*, *Defluviitaleaceae*, *Defluviitaleaceae_UCG-011*, *norank_o__Oscillospirales*, and *Alistipes* (Fig. 5F and G).

Six genera of bacteria were significantly different among the groups. Relative abundances of *Clostridium_sensu_stricto_1* (Fig. 5H), *Terrisporobacter* (Fig. 5I), and *Lachnospiraceae* (Fig. 5K) in the MSCo group increased significantly compared with those in the CCo group, whereas the relative abundance of *Lactobacillus* decreased significantly (Fig. 5J). The relative abundance of *Phascolarctobacterium* (Fig. 5L) in the PMSCo group increased significantly compared with that in the CCo group, whereas that of *Lactobacillus* decreased significantly (Fig. 5J). The relative abundance of the *Rikenellaceae_RC9_gut_group* (Fig. 5M) in the PMSCo group increased significantly compared with that in MSCo group.

### PICRUSt predictions of intestinal microbe functions.

**FIG 6:**
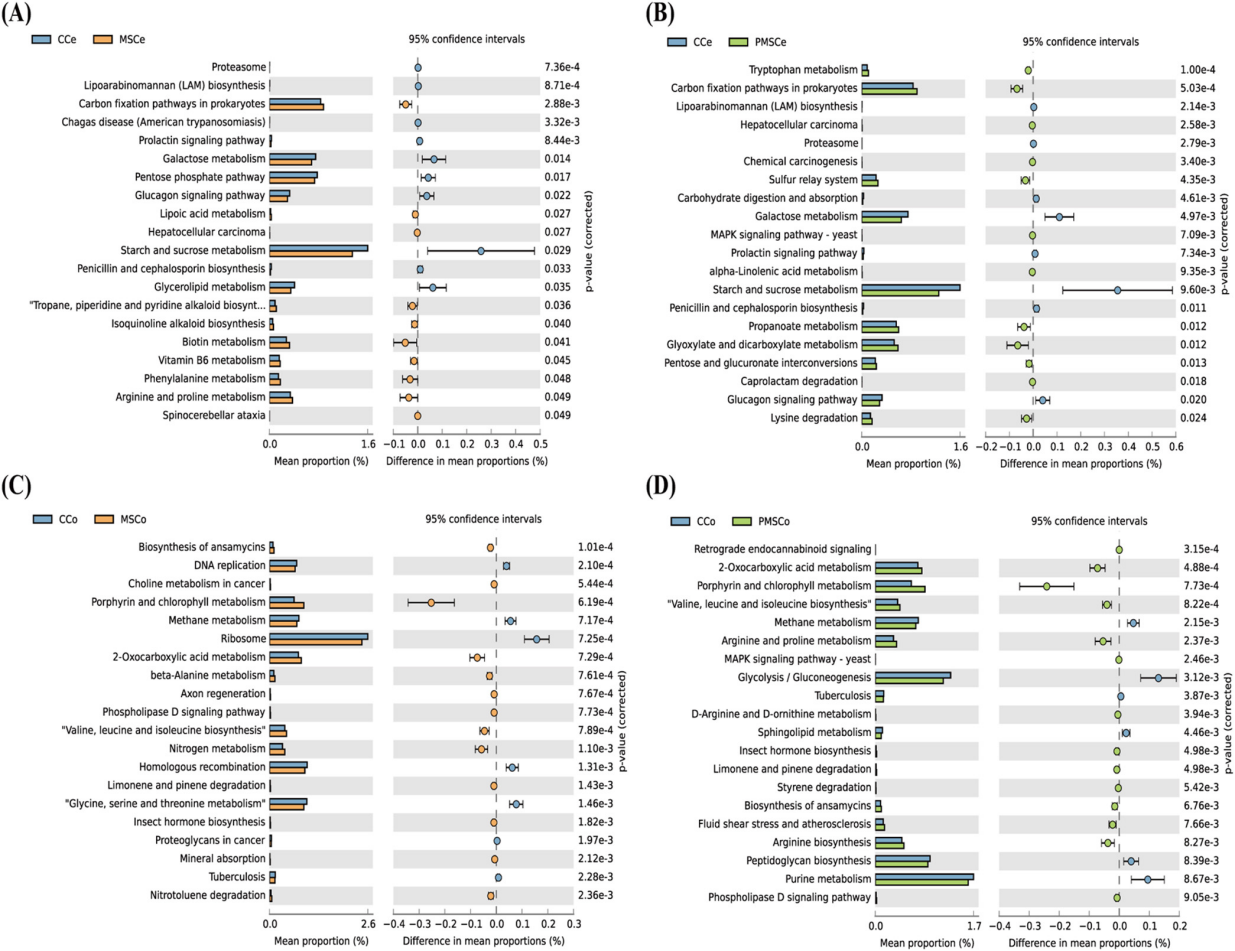
Prediction of metabolic pathways regulated by intestinal microbes. (A) Top 20 metabolic pathways with the most significant differences between CCe and MSCe; (B) top 20 metabolic pathways with the most significant differences between CCe and PMSCe; (C) top 20 metabolic pathways with the most significant differences between CCo and MSCo; (D) top 20 metabolic pathways with the most significant differences between CCo and PMSCo. CCe, cecum of the control; MSCe, cecum of the mulberry silage group; PMSCe, cecum of the paper mulberry silage group; CCo, colon of the control; MSCo, colon of the mulberry silage group; PMSCo, colon of the paper mulberry silage group.

To predict functions of the intestinal microbes, PICRUSt predictions of function were obtained based on the KEGG database, and STAMP software was used to select the 20 metabolic pathways with the most significant differences. Analysis of cecum microbes indicated that 10 metabolic pathways increased significantly in the MSCe group compared with the CCe group, including those of vitamin B_6_ metabolism, phenylalanine metabolism, and arginine and proline metabolism, and that 10 metabolic pathways decreased significantly in the MSCe group compared with the CCe group, including those of galactose metabolism, pentose phosphate, and starch and sucrose metabolism (Fig. 6A). Twelve metabolic pathways increased significantly in the PMSCe group compared with the CCe group, including those of propanoate metabolism, pentose and glucuronate interconversions, and lysine degradation, whereas 8 metabolic pathways decreased significantly in the PMSCe group compared with the CCe group, including those of carbohydrate digestion and absorption, galactose metabolism, and starch and sucrose metabolism (Fig. 6B).

Analysis of colon microbes indicated that 13 metabolic pathways increased significantly in the MSCo group compared with the CCo group, including those of porphyrin and chlorophyll metabolism, nitrogen metabolism, and nitrotoluene degradation, and that 7 metabolic pathways decreased significantly in the MSCo group compared with the CCo group, including those of DNA replication, methane metabolism, and glycine, serine, and threonine metabolism (Fig. 6C). Fourteen metabolic pathways increased significantly in the PMSCo group compared with the CCo group, including those of 2-oxocarboxylic acid metabolism, porphyrin and chlorophyll metabolism, and arginine and proline metabolism, and 6 metabolic pathways decreased significantly in the PMSCo group compared with the CCo group, including those of methane metabolism, glycolysis/gluconeogenesis, and peptidoglycan biosynthesis (Fig. 6D).

### Correlation analysis between intestinal microbes and short-chain fatty acids and longissimus dorsi muscle fatty acids.

**FIG 7:**
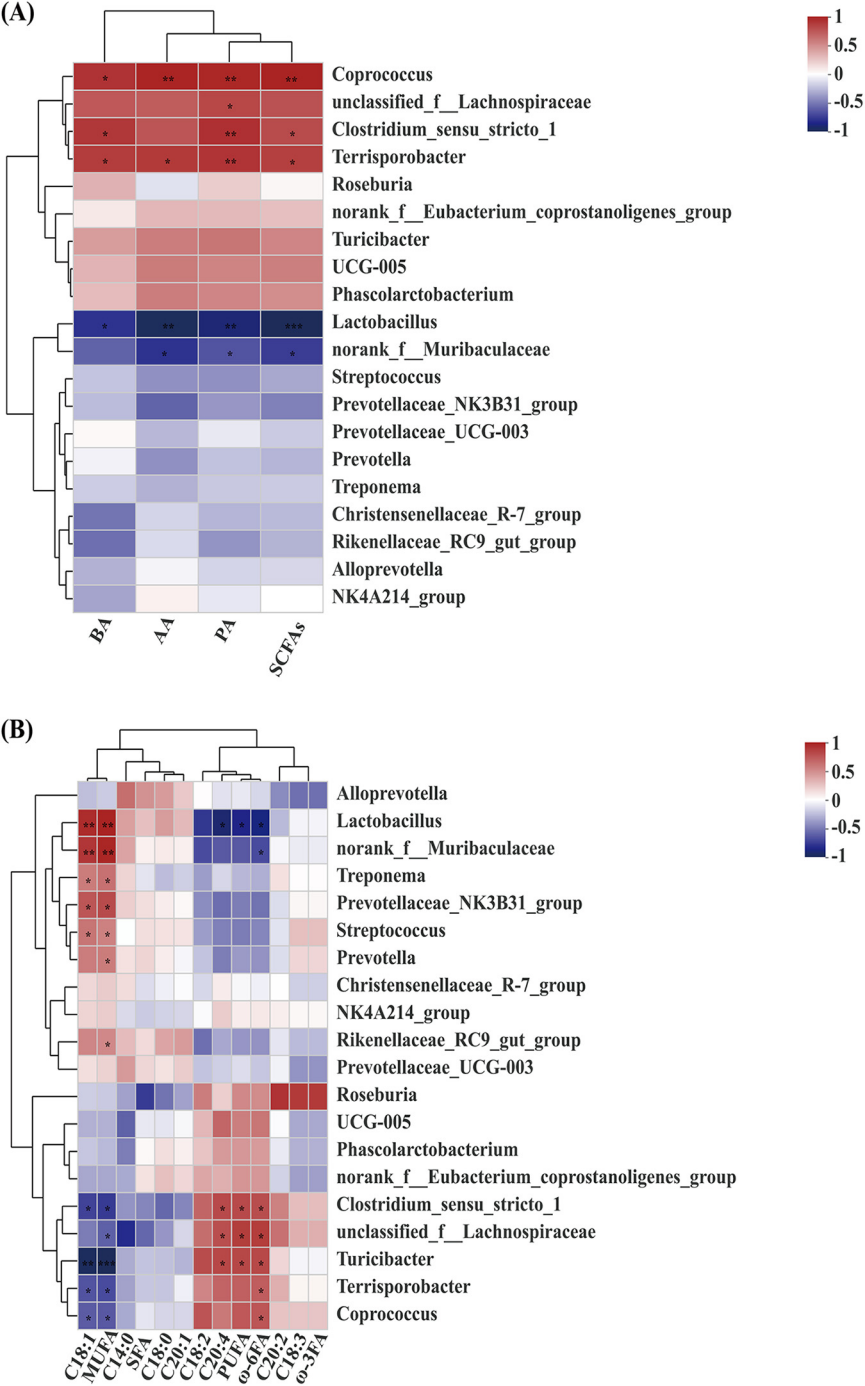
Correlation analysis of intestinal microbes with short-chain fatty acids and longissimus dorsi muscle fatty acids. (A) Correlation analysis of intestinal microbes with short-chain fatty acids; (B) correlation analysis of intestinal microbes with longissimus dorsi muscle fatty acids. The *x* axis presents environmental factors, and correlation coefficients (*r*) and *P* values were obtained by calculation. Coefficients are displayed in different colors as indicated by the legend on the right, with red representing a positive correlation and blue representing a negative correlation. *P* values were adjusted by FDR using the Benjamini-Hochberg method (FDR < 0.05). *, 0.01 < *P* < 0.05; **, *P* < 0.01; ***, *P* < 0.001.

Pearson correlation analysis was used to determine the correlations between SCFAs and colon microbes. A metabolic association heat map indicated positive or negative correlations between SCFAs and microbes. *Coprococcus* and *Terrisporobacter* were positively correlated with BA, AA, PA, and total SCFAs. *Unclassifed_f__lachnospiraceae* was positively correlated with PA. *Clostridium_sensu_stricto_1* was positively correlated with BA, PA, and total SCFAs. *Lactobacillus* was negatively correlated with AA, PA, BA, and total SCFAs, and *norank_f__Muribaculaceae* was negatively correlated with AA, PA, and total SCFAs (Fig. 7A). The results indicated that those microbes had important interactions with SCFAs.

Pearson correlations were also examined between fatty acids of the longissimus dorsi muscle and colon microbes. *Lactobacillus*, *norank_f__Muribaculaceae*, Treponema, *Prevotellaceae_NK3B31_group*, and Streptococcus were positively correlated with oleic acid (C_18:1_), whereas *Clostridium_sensu_stricto_1*, *Turicibacter*, *Terrisporobacter*, and *Coprococcus* were negatively correlated with oleic acid (C_18:1_). *Lactobacillus*, *norank_f__Muribaculaceae*, Treponema, *Prevotellaceae_NK3B31_group*, Streptococcus, *Prevotella*, and *Rikenellaceae_RC9_gut_group* were positively correlated with MUFA, whereas *Clostridium_sensu_stricto_1*, *unclassified_f_Lachnospiraceae*, *Turicibacter*, *Terrisporobacter*, and *Coprococcus* were negatively correlated with MUFA. *Clostridium_sensu_stricto_1*, *unclassified_f__Lachnospiraceae*, and *Turicibacter* were positively correlated with arachidonic acid (C_20:4_), while *Lactobacillus* was negatively correlated with arachidonic acid (C_20:4_). *Clostridium_sensu_stricto_1*, *unclassified_f__Lachnospiraceae*, and *Turicibacter* were positively correlated with PUFA, whereas *Lactobacillus* was negatively correlated with PUFA. *Clostridium_sensu_stricto_1*, *unclassified_f__Lachnospiraceae*, *Turicibacter*, *Terrisporobacter*, and *Coprococcus* were positively correlated with ω-6FA, but *Lactobacillus* and *norank_f__Muribaculaceae* were negatively correlated with ω-6FA (Fig. 7B). These results suggested that those microbes were important in the production of fatty acids in the longissimus dorsi.

## DISCUSSION

### Effects of different silage diets on growth performance of finishing pigs.

In recent years, silage feed has been increasingly provided to finishing pigs. Weight gain and FCR improve in finishing pigs fed clover silage (19), although there are no significant differences in ADG and FCR among treatments when finishing pigs are fed diets with 3% rye silage (20). Mulberry trees are rich in alkaloids, polyphenols, flavonoids, and anthocyanins, which are very beneficial for animal health (5), and leaves of paper mulberry are rich in alkaloids and flavonoids, which have high medicinal and nutritional value (12). In this study, mulberry and paper mulberry were silage processed and added to the diet of finishing pigs. The ADG and FCR were not significantly different between silage treatment groups and the control, which is a result not consistent with that in piglets (12). The difference in effects of silage may be because different growth stages of pigs have different nutritional requirements (21).

### Effects of different silages on the meat quality of finishing pigs.

The physicochemical properties of pork, such as water holding capacity, drip loss, meat color, and marbling, and nutrients such as fatty acids are used to evaluate meat quality. The quality of pork is affected by many factors, including heredity, environment, and diet, but animal feed is a factor that directly affects meat quality (22). The overall acceptability of meat color, appearance, marbling score, and fresh meat sensory score of pigs fed silage is higher than that of commercial feed (23). In this study, the values of L* and a* indicted the best meat color was in the mulberry silage group. The water holding capacity of meat with paper mulberry silage in the diet was significantly higher than that of the control, while that in the mulberry silage group tended to be higher. The marbling scores of meat in the mulberry and paper mulberry silage groups were both significantly higher than that of the control. Effects on muscle water holding capacity are mainly due to the antioxidant capacity of muscle (24). Mulberry silage includes antioxidant flavonoids and polyphenols (5), and paper mulberry silage is rich in antioxidant polyphenols (25). The high levels of antioxidants might explain why water holding capacity in the silage groups was higher (better) than that in the control. Deposition of intramuscular fat directly affects the marbling score, and an increase in marbling affects the tenderness of pork (26). With increases in marbling, the edible quality gradually improves. However, too much fat has adverse effects on human health, and thus, the content of fat in pork should be controlled in a reasonable range of 1.5% to 2.5% (27). The results indicated that silage feed can improve the water holding capacity and intramuscular fat deposition of pork.

In addition to the physical properties, fatty acid composition also affects the quality of pork. Differences in diets can affect the production or deposition of fatty acids in pork. Compared with pigs fed conventional feed, pigs fed a red clover silage diet have relatively low levels of SFA and MUFA and relatively high levels of PUFAs, including ω-6FA and ω-3FA (28). In the mulberry silage group, amounts of oleic acid (C_18:1_), MUFA, stearic acid (C_18:0_), SFA, myristic acid (C_14:0_), and arachidonic acid (C_20:1_) decreased, whereas amounts of ω-3FA, α-linolenic acid (C_18:3_), arachidonic acid (C_20:2_ and C_20:4_), linoleic acid (C_18:2_), PUFA, and ω-6FA increased. The α-linolenic acid (C_18:3_) is the precursor of long-chain ω-3 fatty acids, which have a wide range of anti-inflammatory and cardioprotective effects (29). The ω-3FAs are most beneficial to human health and can reduce cholesterol and triglyceride contents in plasma and help prevent cardiovascular diseases (30). In animals, α-linolenic acid (C_18:3_), linoleic acid (C_18:2_), and arachidonic acid (C_20:4_) are essential fatty acids that can only be obtained from food. Polyunsaturated fatty acids are beneficial to human health and meet the needs of consumers (31). They can also increase the activity of T cells and help to improve the immune system and reduce the occurrence of inflammation and tumors (32). Therefore, silage diets had important effects on the nutritional value of pork by affecting the fatty acid composition.

### Effects of different silage treatments on intestinal microbes and associated metabolites.

Short-chain fatty acids are the final products of fermentation of undigested substances in the hindgut and primarily include AA, PA, and BA (33). In the intestine, SCFAs are rapidly absorbed. Propionic acid is mainly used in the gluconeogenesis pathway, whereas AA can promote the synthesis of fatty acids and cholesterol (34). Butyric acid is primarily absorbed by intestinal epithelial cells and is the main energy source for metabolism of hindgut cells (35). In this study, contents of AA, PA, BA, and total SCFAs in the colon were higher in the silage groups than in the control. Short-chain fatty acids are the main metabolites of intestinal microbial fermentation. The 16S rRNA sequencing in the present study indicated that abundance of *Proteobacteria* at the phylum level was higher in the mulberry and paper mulberry silage groups than in the control, with the difference significant in the paper mulberry silage group. *Proteobacteria* is the most unstable phylum in the intestinal tract and is easily influenced by environmental factors such as diet, but it can also promote the production of lactic acid (36). At the genus level in the cecum, the phylum *Christensenellaceae_R-7_group* might be important as both core and differential bacteria in the paper mulberry silage group. *Christensenellaceae_R-7_group* belongs to *Firmicutes* and can produce SCFAs (37). *Christensenellaceae_R-7_group* is very important to the structure and function of the host intestinal tract (38) At the genus level in the colon, *Clostridium_sensu_stricto_1*, *Terrisporobacter*, and *Lachnospiraceae* were both core and differential bacteria of the mulberry silage group and therefore may have important roles. *Clostridium_sensu_stricto_1* is a fiber-degrading bacterium that can degrade cellulose and hemicellulose by using glucose (39) and is also associated with the formation of SCFAs (40). Correlations between intestinal microbes and SCFAs were also evaluated. *Clostridium_sensu_stricto_1* was significantly positively correlated with the production of PA, BA, and SCFAs. *Terrisporobacter* can generate lactic acid and synthesize BA by exploiting AA and lactic acid under anaerobic conditions, which prevents the accumulation of lactic acid to stabilize the intestinal environment (41). In this study, *Terrisporobacter* was significantly positively correlated with the production of AA, BA, PA, and SCFAs. *Lachnospiraceae* is an obligate anaerobic bacterium that is abundant in the human intestine, and it can resist the colonization of drug-resistant pathogens by converting primary bile acids into secondary bile acids and producing SCFAs (42). Moreover, *Lachnospiraceae* can convert butyrate to propionate on different substrates (43). In this study, *Lachnospiraceae* was significantly positively correlated with the production of PA. The functions of *Clostridium_sensu_stricto_1*, *Terrisporobacter*, and *Lachnospiraceae* explained why amounts of AA, PA, BA, and SCFAs were highest in the different diet groups. In addition, there was a significant increase of *Phascolarctobacterium* in the paper mulberry silage group of the colon compared with the control group. *Phascolarctobacterium* is one kind of asaccharolytic, succinate-utilizing bacterium (44), and propionic acid is the main end product of succinate fermentation (45). This may be one reason why the contents of acetic acid, propionic acid, and SCFAs increased significantly in the paper mulberry silage group. The *Rikenellaceae_RC9_gut_group* can produce propionate and acetate as fermentation end products (46). *Lactobacillus* bacteria can produce lactic acid from the fermentation of glucose or lactose (47). After silage, glucose content in feed is low (48). Therefore, when feeding silage to finishing pigs, high *Lactobacillus* abundance is not needed to metabolize glucose, which may explain why *Lactobacillus* abundance was higher in the control than in both silage groups. The correlation analysis between microbes and SCFAs further demonstrated that *Coprococcus*, as the main butyric acid-producing bacterium (49), is positively correlated with butyric acid, acetic acid, and propionic acid, possibly because acetic acid can be used as the substrate for butyric acid synthesis (50) and *Coprococcuscatus* can convert lactic acid into propionic acid through the acrylate pathway (51). Microbes function in host metabolism, and therefore, predictions of function by PICRUSt were analyzed by STAMP software. The results indirectly indicated that most of the microbes in the control group were involved probably in galactose, pentose phosphate, starch, sucrose, and glycolysis metabolism, while most of the microbes in the two silage groups were involved in vitamin B_6_, phenylalanine, arginine and proline, and propanoate metabolism and lysine degradation. The results demonstrated that galactose metabolism, starch and sucrose metabolism, carbohydrate digestion, and absorption significantly decreased in the silage feed treatment group, while they increased in the control group. Previous reports have shown that some protein and soluble sugar have been degraded during silage production (48, 52). In this study, the PICRUSt functional analysis of intestinal microbes also indicated that some proteins and soluble saccharides were most likely degraded during silage production.

### Potential mechanism to improving meat quality.

Diet is important in determining tissue fatty acid content (53). Semova et al. reported that as the absorption rate of fatty acids in a diet increases the number and volume of fat droplets in intestinal epithelial cells and fat deposition increase (54). In addition, SCFAs produced by microbial fermentation can regulate the expression of genes related to fat metabolism. Acetic acid can promote the synthesis of fatty acids and cholesterol (55), whereas PA can inhibit AA from producing cholesterol and fat in the liver (56). Short-chain fatty acids also have effects on fat storage (57). Therefore, SCFAs produced by intestinal microbial fermentation may directly regulate the expression of genes associated with lipid metabolism or act as signal molecules to regulate the production of intramuscular fat. Correlations between intestinal microbes and fatty acids also indicated that *Clostridium_sensu_stricto_1* promoted the accumulation of arachidonic acid (C_20:4_), PUFA, and ω-6FA and inhibited the accumulation of oleic acid (C_18:1_) and MUFA. In addition, correlations indicated that *Terrisporobacter* promoted the accumulation of ω-6FA and inhibited the accumulation of oleic acid (C_18:1_) and MUFA. *Lachnospiraceae* promoted the accumulation of arachidonic acid (C_20:4_), PUFA, and ω-6FA and inhibited the accumulation of MUFA. In pigs, the driving force has been to increase with a higher ratio of PUFA to saturated fatty acids to produce healthier meat, which can be achieved by feeding (58). It can be seen that *Clostridium_sensu_stricto_1*, *Terrisporobacter*, and *Lachnospiraceae* acting as SCFA-producing bacteria were also closely related to meat fatty acids. Overall, feeding finishing pigs silage feed in this study, especially mulberry silage, improved meat quality by altering intestinal microbial communities to increase the abundance of SCFA-producing and beneficial microbes. The resulting changes in bacteria can alter the content of intestinal SCFAs, which can affect the generation and deposition of muscle fatty acids. The study provides a theoretical basis for the application of mulberry silage in finishing pigs and identifies a new strategy to improve meat quality.

## MATERIALS AND METHODS

### Ethics approval.

The animal study was approved by the Institutional Animal Ethics Committee of Henan Agricultural University (approval HENAU-2020-013).

### Animal experiment.

A total of 240 healthy finishing pigs (Duroc × Landrace × Yorkshire) with an average body weight of 60 kg were fed the same diet and in the same house before the start of the experiment. The 240 pigs were then randomly separated into three treatments: control group, 10% mulberry silage (MS) group, and 10% paper mulberry silage (PMS) group. Pigs continued to be fed in the same house. The amount added was based on dry matter. Each treatment had 4 replicates, and each replicate included 20 finishing pigs. Finishing pigs were fed two times per day (8:00 a.m. and 5:00 p.m.) and had free access to diet and water during the experiment. The experiment lasted for 61 days, including a 7-day preexperiment period and a 54-day feeding experiment. After the feeding experiment, one finishing pig was randomly selected from each replicate for slaughter (i.e., 4 pigs per treatment [*n *= 4]). Samples from the pigs were analyzed for meat quality, intestinal SCFAs, and microbiota. Methods and procedures were approved by the Professional Committee of Animal Welfare Ethics of Henan Agricultural University. All animal treatments and experiments were performed according to the recommendations of the guidelines for ethical review of animal welfare in the national standards of the People’s Republic of China. Mulberry and paper mulberry silages were provided by Henan Shi ji tian yuan Ecological Science and Technology Co., Ltd., and Lan kao Zhong ke hua gou Biological Technology Co., Ltd., respectively. The experimental diet was designed according to the nutrition standard of the NRC of the United States, and the diet formula and nutritional composition are shown in Table 3.

**TABLE 3:** Ingredient composition and nutritional level in different diets

Parameter	Result for^*a*^:		
	CON	MS	PMS
Ingredient (%)			
Corn	72.41	67.33	68.55
Soybean meal	18.12	18.80	17.55
Soybean oil	0	1.90	1.90
Bran	7.2	0.04	0
Calcium hydrogen phosphate	0.70	0.52	0.53
Limestone powder	0.36	0.25	0.18
Fermented mulberry	0	10.00	0
Fermented paper mulberry	0	0	10.00
1% premix^*b*^	1.00	1.00	1.00
Lysine (98% pure)^*c*^	0.21	0.16	0.29
Total	100.00	100.00	100.00
Nutrient composition			
Digestive energy (MJ/kg)	13.67	13.67	13.67
Crude protein (%)	15.19	15.24	15.25
Ether extract (%)	4.47	6.09	6.11
Neutral detergent fiber (%)	12.24	13.15	14.99
Acid detergent fiber (%)	5.13	7.23	8.22
Ca (%)	0.51	0.51	0.51
Total phosphorus (%)	0.49	0.44	0.43
Available phosphorus (%)	0.24	0.24	0.24
Lys (%)	0.95	0.94	0.95

a CON, control group; MS, mulberry silage group; PMS, paper mulberry silage group.

b The 1% premix provided the following dietary supplements per kilogram: vitamin A, 5,000 IU; vitamin D_3_, 3,000 IU; vitamin E, 40.1 IU; vitamin B_2_, 23.2 mg; vitamin B_1_, 20.01 mg; nicotinic acid, 16 mg; pantothenic acid, 10 mg; biotin, 0.168 mg; folacin, 1.28 mg; Cu, 11.2 mg; Fe, 110 mg; Zn, 65.6 mg; Mn, 37.6 mg; I, 0.47 mg; Se, 0.30 mg.

c The purity of the lysine added in feed is 98%.

### Growth performance of finishing pigs.

The following measures of growth performance were calculated: (i) average daily feed intake (ADFI) = average daily supplement (kg) − average daily surplus (kg), (ii) average daily gain (ADG) = (average final weight − average initial weight)/test days, and (iii) feed conversion rate (FCR) = ADFI/ADG.

### Determination of the physical characteristics of pork.

Approximately 500 g of longissimus dorsi muscle was collected, and meat color, marbling, drip loss, and water holding capacity were measured. Meat color parameters included redness (a*), yellowness (b*), and lightness (L*) and were determined by a portable chromameter at 45 min after slaughter. The L* value is the lightness coefficient, and the closer the value is to 100, the brighter the sample. The a* value represents red-green, and the b* value represents yellow-blue. When the a* value is positive, the color is reddish, and when a* value is negative, the color is greenish. When the b* value is positive, the color is yellowish, and when b* value is negative, the color is bluish. Meat marbling was observed by cross section of the longissimus dorsi muscle at the junction of the last thoracic vertebra and the first lumbar vertebra. The scale of marbling score was 1 to 5 (1 = devoid to practically devoid, 2 = traces to slight, 3 = small to modest, 4 = moderate to slightly abundant, and 5 = moderately abundant or greater). Marbling was observed 45 min later after slaughter. All samples were analyzed by five people, and the evaluators did not know the source of the samples. Water holding capacity pressure was measured by a weight pressurization method. In brief, longissimus dorsi muscle from the last lumbar to the first thoracic vertebra was cut into 1-cm-thick slices, and the slices were weighed as m1 values. Then, pork samples were treated in the following order: filter paper (18 layers), gauze (5 layers), pork sample, gauze (5 layers), and filter paper (18 layers). An iron weight (30 kg) was placed on top, and after 5 min, samples were weighed as m2 values. The following formulas were used to calculate water holding capacity: (i) water loss rate = [(m1 − m2)/m1] × 100% and (ii) water holding capacity = 1 − (water loss rate/moisture content of the pork samples). Drip loss was measured by a hanging method that relied only on gravity within 2 h after slaughter. Longissimus dorsi muscle samples of 5 cm (length) by 3 cm (width) by 2 cm (thickness) were weighed as m1 values. One end of a pork sample was hooked with a thin wire so that the muscle fibers were oriented vertically downward and hung in a Ziploc bag for 24 h in a refrigerator at 4°C. Samples were then weighed as m2 values. The following formula was used to calculate drip loss: drip loss = [(m1 − m2)/m1] × 100%.

### Determination of fatty acids in muscle.

Fatty acids were determined in 100-g samples of longissimus dorsi muscle by gas chromatography (GC). The GC conditions were set according to a previous study as follows: injection volume, 1 μL; split ratio, 1:15; initial temperature of 150°C ramped to 240°C at 4°C/min; and nitrogen flow rate, 40 mL/cm^2^.

### Determination of short-chain fatty acids in the colon.

Collected colon chyme was diluted with distilled water (mass/volume ratio of 1:1). After shaking and mixing, samples were centrifuged at 12,000 × *g* for 10 min. To 1 mL of supernatant, 0.2 mL of 25% metaphosphoric acid was added. After 30 min, samples were centrifuged at 12,000 × *g* for 10 min. To 100 μL of supernatant, 100 μL of methanol was added, and following thorough mixing, samples were centrifuged at 12,000 × *g* for 10 min. Supernatants were collected, and GC was used to determine amounts of SCFAs in the colon. Samples were analyzed on an HP-88 column and separated using a Trace 1310 GC with a flame ionization detector. Samples were run with a split ratio of 20:1 and a column flow of 1.3 mL/min. Hydrogen was used as the carrier gas. The injector and detector temperatures were 270°C and 290°C, respectively, according to a previous study. The calibration curve constructed by GC-mass spectrometry (MS) data of corresponding SCFA standards was used to calculate the concentration of SCFA in colon samples.

### DNA extraction and 16S rRNA gene sequencing.

After cecum and colon chyme were collected and sorted, samples were sent to Shanghai Meiji Biomedical Technology Co., Ltd., for sequencing of bacterial DNA. The bacterial DNA of cecum and colon was extracted by an E.Z.N.A. soil DNA kit (Omega Bio-Tek, Norcross, GA, USA) according to the manufacturer’s protocols. Concentration and purity of DNA were measured by a NanoDrop 2000 UV-visible (UV-vis) spectrophotometer (Thermo Scientific, Wilmington, DE, USA), and DNA quality was verified by 1% agarose gel electrophoresis. The upstream primer 338F (5′-ACTCCTACGGGAGGCAGCAG-3′) and the downstream primer 806R (5′-GGACTACHVGGGTWTCTAAT-3′) were used to amplify the V3-V4 hypervariable region of the 16S rRNA gene by PCR. The PCR program was the following: 3 min of denaturation at 95°C, followed by 27 cycles of 30 s at 95°C, 30 s of annealing at 55°C, and 45 s of elongation at 72°C, with a final extension at 72°C for 10 min. In the PCR system, each 20-μL reaction mixture contained 4 μL of 5× FastPfu buffer, 2 μL of 2.5 mM deoxynucleoside triphosphates, 0.8 μL of each primer (5 μM), 0.4 μL of FastPfu polymerase, and 10 ng of template DNA. The PCR products were extracted from a 2% agarose gel and further purified using an AxyPrep DNA gel extraction kit (Axygen Biosciences, Union City, CA, USA). Three independent PCRs from each sample were conducted, and the PCR products were mixed and sequenced as a single sample (59). A TruSeqIM DNA sample prep kit was used to build the library, and the MiSeq PE300 platform of the Illumina Company was used for sequencing.

### Bioinformatics analysis of sequencing data.

Cecum and colon samples were collected and sequenced by Shanghai Magi Biomedical Technology Co., Ltd. Raw fastq files were demultiplexed, quality filtered using Trimmomatic, and merged using FLASH. In that process, (i) reads were truncated at any site receiving an average quality score of <20 over a 50-bp sliding window, (ii) primers were exactly matched, allowing 2-nucleotide mismatching, and reads containing ambiguous bases were removed, and (iii) sequences with overlap longer than 10 bp were merged according to the overlap sequence. Operational taxonomic units (OTUs) with 97% similarity cutoff were clustered using UPARSE (v7.1 [http://drive5.com/uparse/]), and chimeric sequences were identified and removed. The taxonomy of each OTU representative sequence was analyzed by RDP Classifier (http://rdp.cme.msu.edu/) against a 16S rRNA database (i.e., Silva SSU128) using a confidence threshold of 0.7.

### Microbial diversity analysis.

Sample biodiversity was calculated using Chao 1 and Shannon indices and then applying a Wilcoxon rank sum test. Changes in relative abundances of bacteria are shown using column charts. The weighted Unifrac was used in a principal-coordinate analysis (PCoA). Hierarchical cluster analysis of Bray-Curtis samples based on OTU level was used to summarize the composition of cecum and colon bacterial communities. The Shannon and Chao indices measure diversity and richness of bacteria, respectively.

### Screening of microbial markers.

The propensity score matching method in the R language was adopted. After matching of propensity scores, DESeq2 was used to screen preliminary differences in abundance of OTUs between groups at the genus level. The ggploT2 package was used to draw a volcano map to show the abundance and significance of differences in OTUs. On the basis of screening abundance differences in OTUs between groups, the linear discriminant analysis (LDA) effect size (LEfSe) method was used to further identify the bacterial markers associated with mulberry and paper mulberry silages. Kruskal-Wallis rank sum tests and Wilcoxon rank sum tests were used to establish a linear discriminant analysis model to screen the biomarkers of each group.

### PICRUSt prediction of microbial functions.

PICRUSt predictions were used to analyze functions of bacteria according to a previous study. To obtain the metabolic pathways with significant differences, STAMP software was used to analyze the significance of PICRUSt predictions at *P* < 0.05 and to visualize the results, according to a previous study.

### Correlation analysis between microbes and short-chain fatty acids and long-chain fatty acids.

To determine the effects of microbes interacting with SCFAs and long-chain fatty acids, redundancy analysis (RDA) was performed at the genus level using the R language vegan packet of Spearman correlation analysis (RDA 2014). Benjamini-Hochberg’s false-discovery rate (FDR) correction was used to correct for multiple testing.

### Statistical analyses.

Growth performance, meat quality, and intestinal SCFA concentrations of finishing pigs were analyzed by SPSS 22.0 software. Data were evaluated by one-way analysis of variance (ANOVA), and differences between mean values were evaluated by Duncan’s test. Significance was set at a *P* value of <0.05. Values are presented as the mean ± standard deviation.

### Data availability.

Raw reads were deposited into the NCBI Sequence Read Archive database under accession no. SRP375531 (https://www.ncbi.nlm.nih.gov/Traces/study/).
