# Supplementary material for: Effects of Silage Diet on Meat Quality through Shaping Gut Microbiota in Finishing Pigs
Source: Microbiol Spectr. 2022 Dec 12;11(1):e02416-22. doi: 10.1128/spectrum.02416-22 (PMC9927310; doi:10.1128/spectrum.02416-22)
Supplement: Supplemental file 1 — Supplemental material. Download spectrum.02416-22-s0001.pdf, PDF file, 0.3 MB [file spectrum.02416-22-s0001.pdf]

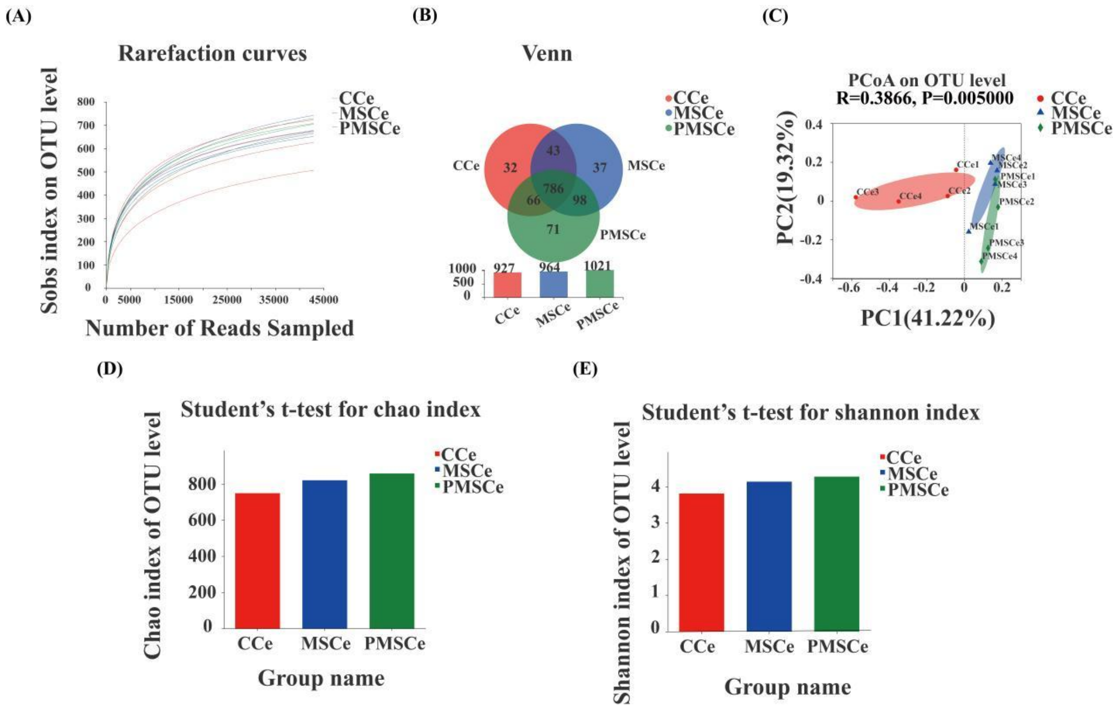

Supplementary Fig.1 Diversity index analysis of cecum microbes. (A) Rarefaction curves. (B) Venn diagram. (C) Principal component analysis using operational taxonomic units (OTU) by Bray-Curtis. (D) Chao index. (E) Shannon index. OTU = operational taxonomic units. Data are presented as means ± SD. *, 0.01 < P < 0.05; **, P < 0.01. CCe, cecum of the control; MSCe, cecum of the mulberry silage group; PMSCe, cecum of the paper mulberry silage group. 4 samples in cecum per treatment, N=4.

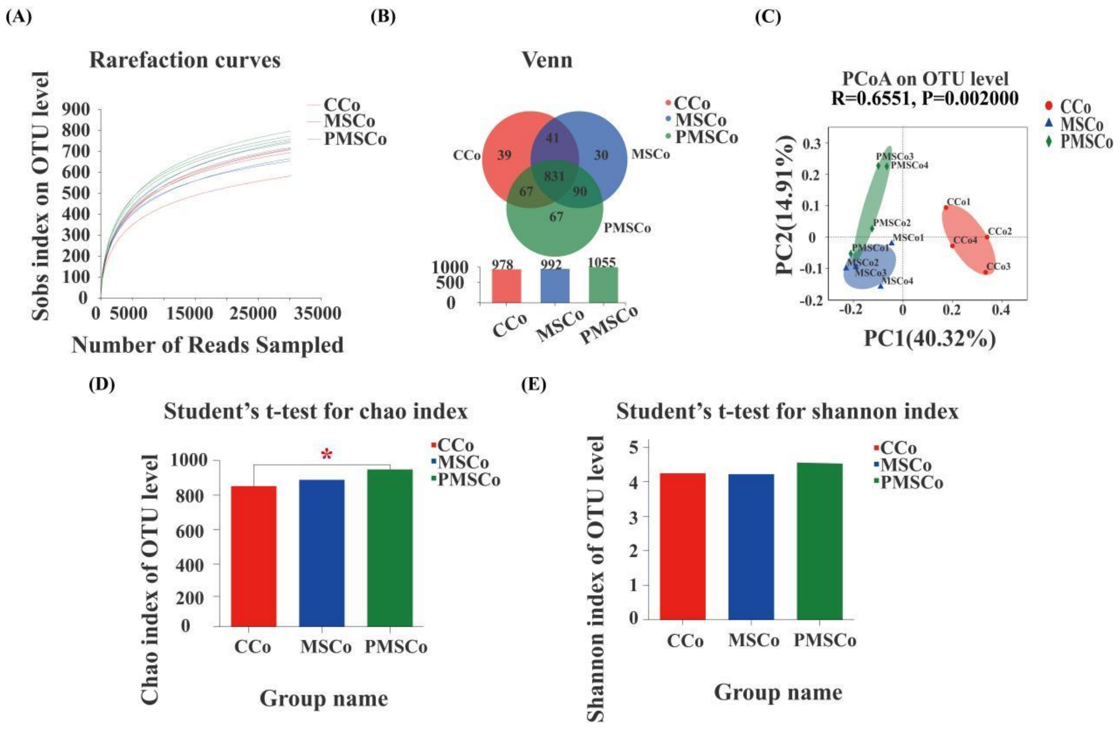

Supplementary Fig.2 Diversity index analysis of colon microbes. (A) Rarefaction curves. (B) Venn diagram. (C) Principal component analysis using operational taxonomic units (OTU) by Bray-Curtis. (D) Chao index. (E) Shannon index. OTU = operational taxonomic units. Data are presented as means ± SD. *, 0.01 < P < 0.05; **, P < 0.01. CCo, colon of the control; MSCo, colon of the mulberry silage group; PMSCo, colon of the paper mulberry silage group. 4 samples in colon per treatment, N=4.
